# Radon Exposure to the General Population of the Fernald Community Cohort

**DOI:** 10.3390/atmos16080939

**Published:** 2025-08-05

**Authors:** John F. Reichard, Swade Barned, Angelico Mendy, Susan M. Pinney

**Affiliations:** 1 Department of Environmental and Public Health Sciences, University of Cincinnati, Cincinnati, OH 45267, USA; 2 EnSafe, 525 Vine Street, Cincinnati, OH 45202, USA

**Keywords:** radon, Fernald, exposure, EPA action limit

## Abstract

The Fernald Feed Materials Production Center (FMPC), located in Fernald, Ohio, USA, released radon (Rn) as a byproduct of the processing of uranium materials during the years from 1951 to 1989. Rn is a colorless, odorless gas that emits charged alpha radiation that interacts with cells in the lung and trachea-bronchial tree, leading to DNA damage, mutations, and tumor initiation. The purpose of this project was to use evidence collected by the Fernald Dosimetry Reconstruction Project and other sources to estimate the outdoor Rn exposure to individuals in the community immediately surrounding the FMPC during the years of plant operation. Using previously tabulated source terms, diffusion and meteorological data, and self-reported detailed residential histories, we estimated radon exposure for approximately 9300 persons who lived at more than 14,000 addresses. The results indicated that a portion of the population cohort experiences mean annual Rn exposure exceeding the U.S. Environmental Protection Agency (EPA) action limit of 4 pCiL^−1^. These exposure estimates support the analysis of the incidence of lung cancer in the Fernald Community Cohort (FCC).

## 1. Introduction

The FMPC in Fernald, Ohio, USA, was a government-owned, contractor-operated facility that converted uranium ore concentrates and materials recycled from nuclear weapons production to either uranium oxides or metal ingots [1]. During its years of operation, the Fernald FMPC emitted large amounts of radioactive nuclides, including Rn gas, which likely affected the health of plant workers and potentially that of the surrounding community members. The objective of this project was to use evidence collected by the Fernald Dosimetry Reconstruction Project and other sources to estimate the external exposure of the nearby community to Rn gas emitted by the plant during its years of operation.

The FMPC was located in the rural community of Fernald, which is about 25 km northwest of Cincinnati, Ohio, and was one of several sites where the US Department of Energy processed uranium ore and recycled uranium material received from other facilities [1]. The plant operated from 1951 through to 1988, and during that time, the nearby community more than doubled in population [2]. The plant used high-temperature metal digestion to extract uranium from pitchblende or other uranium ores and recycled uranium-containing metal products. The raffinate waste (a slurry of uranium mill tailings) that remains after uranium extraction is a radioactive waste material, locally called K-65, which contains very high concentrations of radium-226 (^226^Ra) that decays to form radon-222 (Rn) as a daughter nuclide. This K-65 material was, by far, the major source of Rn emitted from the FMPC facility; however, other minor sources, including waste metal oxide material, also contributed to the total amount of Rn released from the plant [3] (pp. 28–31).

**Figure 1.**
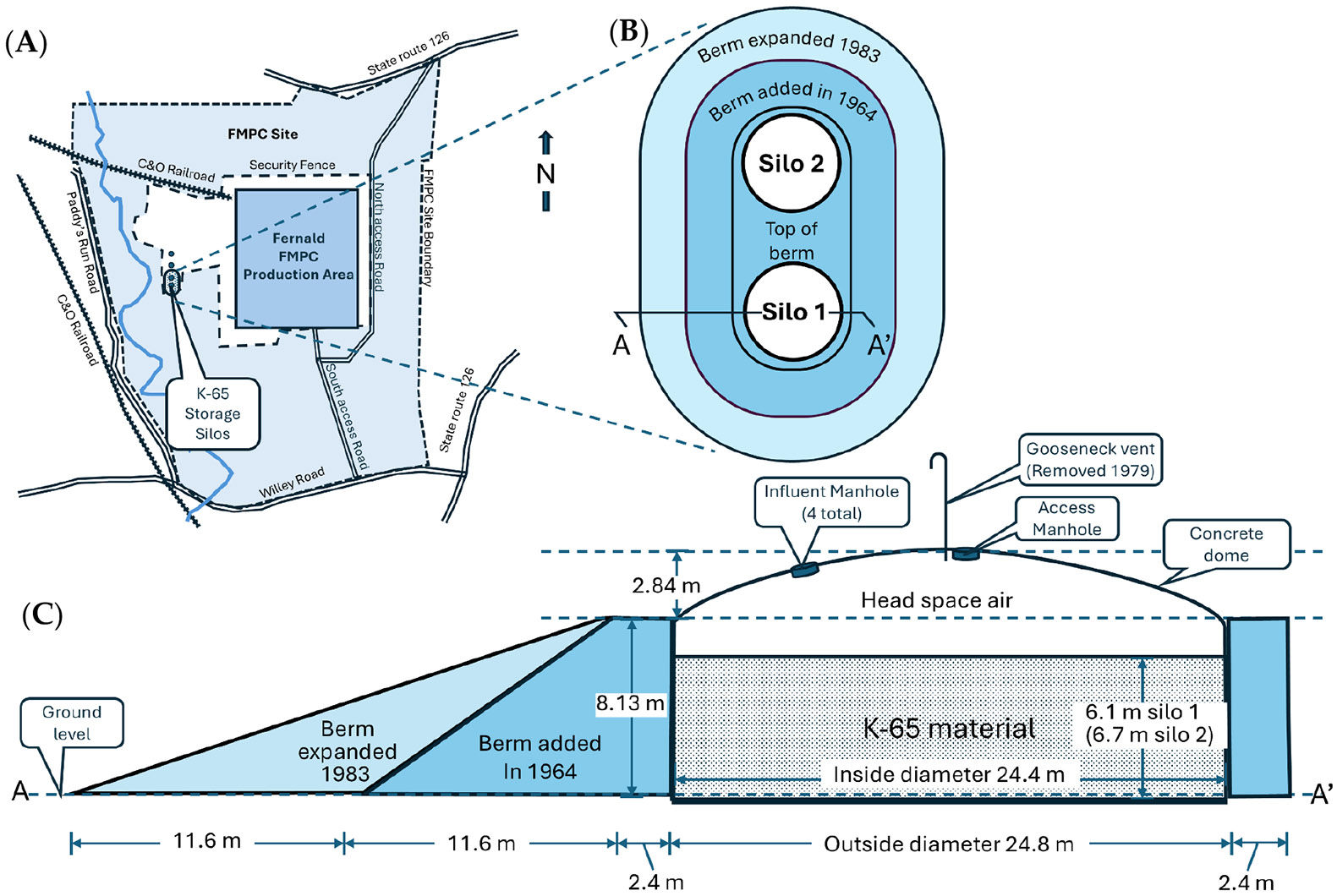
Fernald FMPC K-65 waste storage silos. (**A**). Location of the storage silos relative to the FMPC production facility. (**B**) Layout of the two K-65 silos and surrounding earthen berms that were added after silo construction. (**C**). Cross section of silo 1 with associated measurements and structural details. This figure is adapted from figures J-1, J-11, and J-12 in [3].

K-65 material was stored at the FMPC even before uranium processing operations began. From September 1951 to July 1952, 13,000 208-L (55-US-gallon) drums, each containing 228 kg of K-65 waste, were transferred to the facility from other US facilities. During this time frame, four concrete thank-like silos were constructed, two of which were dedicated to the long-term storage of K-65 material; a third silo was used for metal oxide material and the fourth was not used and contained only a small quantity of water and very low levels of radioactive materials [3] (p. 28). The two K-65 storage silos were located about 300 m (1000 ft) west of the production facilities near the western fence line of the plant area (Figure 1A) [3]. Each silo was 24 m (80 ft) wide with walls 8.1 m (26 ft) high, with a total storage volume of about 3800 m^3^ (134,000 ft^3^), not including headspace [2,3] (Figure 1C).

The K-65 storage silos were full almost from the start of plant operations. Originally, these silos were not sealed, which allowed Rn-gas to readily escape into the atmosphere through from 1952 to 1979 [1]. After the construction of the silos was completed, most of the drummed K-65 material was transferred into Silo 1. By June 1953, Silo 1 was full, and Silo 2 became operational with the storage site for the remaining drummed K-65 material [2,3]. Uranium processing operations at FMPC began in 1952, and the resulting K-65 raffinate was added to Silo 2, along with K-65 waste that was still arriving from Mallinckrodt Chemical Works facility in St. Louis. In September 1958, Silo 2 was deemed to be full and removed from service [2,3]. In 1972, the amount of K-65 material was measured and determined to be 2860 m^3^ (1.01 × 10^5^ ft^3^) and 3114 m^3^ (1.1 × 10^5^ ft^3^) for Silos 1 and 2, respectively. The metal oxide that was stored in Silo 3 was also measured and determined to have a ^226^Ra content 1/40th that of the K-65 stored in the silos. Thus, for estimating risk, metal oxide stored in Silo 3 and the small Q-11 ore silos within the processing facility are considered insignificant sources of ^222^Rn for community exposure [3] (pp. J-16 and J-17).

Almost from the time of their construction, the silos were deteriorating and continuously leaked Rn gas, which escaped through openings in the domes at the top of the silos and cracks in silo walls [2] (p. 24). Over the years, there were several attempts to mitigate Rn leaks, including two attempts to reinforce the silos with earthen berms (Figure 1B,C); however, the combined efforts only partially reduced emissions but did not stop them. The atmospheric release of Rn from the two silos was significantly reduced when they were capped in 1979. The sequence of events affecting Rn release from the K-65 Silos are presented in Table 1 [2,3]. Despite the known problems with the K-65 silos, no Rn monitoring program was instituted until 1978, when a preliminary radon monitoring effort was initiated using limited number of locations with intermittent Rn data collection. The FMPC established a routine radon monitoring program in July 1980 using six air monitoring stations placed around the facility boundary. Subsequently, studies from 1984 to 1989 measured airborne radon at multiple locations on the plant site and at a few off-site locations at distances up to 2000 m from the silos. Essentially all monitoring data were collected in the period after the silo domes were sealed in 1979 [4] (p. 29). The total quantity of Rn released from Silos 1 and 2 from 1952 through plant closure in 1988 is estimated to be 160,000 Ci (5th and 95th percentiles of 78,000 and 340,000 Ci, respectively) [2] (p. 31). The rate of Rn release from the K-65 silos was greatest during the period from 1959 to 1979 at which time the capped silos released Rn at a rate of approximately 6200 curies per year (Ci y-1) [2] (p. 30). Throughout the period of plant operation, the release of Rn from the K-65 silos, wind patterns, and residential distance were key factors contributing to Rn gas exposures for the communities neighboring the plant.

In 1985, a class action lawsuit was brought against National Lead Company of Ohio, the primary contractor for FMPC operations through the end of 1985, claiming emotional distress to persons who lived near the plant [5]. Operations continued at the site until July of 1989, when all production was halted indefinitely and Fernald was declared an EPA Superfund Site. The Fernald FMPC’s name changed to the Fernald Environmental Management Project in 1991, and the site was prepared for decommissioning, decontamination, and hazardous waste cleanup [5,6].

Rn is a noble gas formed by the radioactive decay of elements in soils and rocks, especially uranium and radium. It is a radioactive element that is released from geologic sites of formation and flows through soils and is released to the atmosphere. The primary human exposure to Rn and its daughter nuclides is inhalation, and numerous studies have linked Rn gas exposure to lung cancer [7]. The radioactive decay of Rn and, more importantly, its short-lived daughter products, emits charged alpha particles that interact with cells and tissues in the lung and trachea-bronchial tree, leading to DNA damage, mutations, and tumor initiation. Therefore, the risk of developing lung cancer increases with cumulative exposure [8]. Public health efforts to mitigate Rn exposure primarily focus on Rn intrusions into homes and other structures through cracks and porous foundation materials; however, Rn from outside the home can be transported by diffusion and air movement through windows, doors, vents, and cracks or openings. The EPA has established an action level of 4 pCi L^−1^ for Rn inside homes but also acknowledges that even Rn levels below 4 pCi·L^−1^ pose some risk, which is consistent with the no-threshold mode of action assumption for genotoxins [9]. It should be noted that the Rn estimations for the FMPC project are not for in-home estimations but rather for concentrations beyond the background amount in ambient air in the Fernald community. However, homes do not provide much protection from outdoor Rn air concentrations, and in-home air concentrations are not very different from outside air concentrations [2,10] (p 49 and p. I-33, respectively).

## 2. Materials and Methods

### 2.1. Dataset of Residential History

The Fernald Medical Monitoring Program was established to provide medical screening for people who lived for at least two consecutive years from 1 January 1952 to 18 December 1984, within a 5-mile (8 km) radius of the FMPC in rural Fernald, Ohio. No workers at the plant were included in this cohort. Screening examinations were conducted between 1990 and 2008 on 9782 community members who enrolled. At enrollment, detailed residential histories were obtained from each person by questionnaire and interview. In addition, detailed information regarding existing health conditions was also collected [11]. Each participant was asked to report every address they lived at during the period from 1951 to 1988 and the time lived at each address (FROM and TO month and year). The reported addresses were verified by asking each cohort member to confirm their residential data at the time of the next yearly questionnaire and to add any incomplete address information. A follow-up phone interview was then conducted with each participant to further clarify residential locations. The exposure domain was limited to a five-mile radius around the FMPC site, which was composed of 5 concentric zones at 1-mile intervals that are divided into 100 sectors of approximately one square mile. The collected residential addresses were then geocoded and mapped to a sector. Each sector corresponded to a cardinal direction from the plant (e.g., north, southwest) and distance, with distance converted to meters.

The total Fernald cohort residential address dataset consists of 14,658 anonymized residential records with beginning and ending dates in a month/year format for each residential location. Since only the month and year were collected for each residential history, not the day, we assumed that residence began on the first day of the month and ended on the last day of the month. Some cohort members were later found to have lived only outside of the exposure domain or had residential history information that could not be geocoded and are not included in this Rn exposure estimation.

### 2.2. Data Corrections and Cleaning

Some cohort members had address dates that predated the participant’s date of birth (DOB). We assumed that the listed residential date corresponded to when a family moved into an address prior to the participant’s birth, because, in many instances, other family members had the same FROM date for that address. In these instances, the DOB rather than the listed residential starting date was used for the start of Rn exposure, assuming the DOB was within the period of plant operation.

Some participants provided residential addresses with FROM or TO dates with unknown months (Table 2, “Residences”). If the month was unknown, 1 July was assumed as the default FROM and 30 June as the default TO date for estimating exposure when not provided by the participant. This is justified because this date is the midpoint of the year, and the probability that the actual residential date occurred before or after this date is equivalent; cumulative errors are likely to be offsetting and provide a similar likelihood of error for overestimating and underestimating residential time. The reliability of this assumption was confirmed by evaluating the distribution of residential dates for all individuals with complete residential dates. From this full dataset analysis, the distribution of when participants move into (residential FROM date) or away from (residential TO date) a property is close to evenly distributed across months of the year, with February, March, November, and December having somewhat fewer arrival or departures and June and July having the most. There were also several participants who provided starting or ending residential dates where the year was missing (Table 2, “Participants”). No assumptions can be made about these records, so addresses without years of residence were excluded from this analysis (Table 2). After adjusting the dataset for these issues, the total number of residential addresses analyzed is 14,658 for a total of 9375 subjects. Of these subjects, 57 additional subjects were removed due to various factors, including missing address information, residential location outside the study area, and the period of residence was outside the period of plant operations (i.e., before 1952 or after 1988). A final total of 9318 subjects and 14,018 total residential locations were evaluated for radon exposure. The data used for this analysis are available at https://med.uc.edu/depart/eh/research/projects/fcc/sharing-of-fcc-data-and-biospecimens.

Because the population demographic data were originally collected using Statistical Analysis System (SAS) software (SAS Institute Inc., 2006, v. 9.1), dates are based on a SAS reference date of 1 January 1960; any date before or after this date is recorded as the number of days greater or less than this date. Using this convention, all dates were calculated in R using the Date () function and the attribute of origin = “1960-01-01”.

The variables that most influenced population Rn exposures from the FMPC were the rate of Rn emission, distance from the emission source, sector location of the residential address relative to the emission source, and prevailing wind direction, speed and stability. Annualized wind frequencies for each octet (N, NE, E, SE, S, SW, W, NW) were evaluated for each of the 100 sectors surrounding the FMPC. Meteorological conditions were assumed, on average, to be similar for each year of plant operations [4] (pp. E-22–E-34). We have not modeled residential indoor or outdoor exposure to decay products from atmospheric Rn emitted by the K-65 silos. Although the decay chain of radon is important for lung cancer risk, based on a half-life of approximately 3.8 days, the decay of Rn contributes only a small decrease in atmospheric levels during the time required for emitted Rn to move across the study area, even at the slowest windspeed used in the model. Furthermore, the large variability of several parameters, including daily and seasonal Rn emission rates, atmospheric conditions (windspeed, directions, and stability), and other input parameters have a much greater impact on the exposure levels to Rn than the loss of Rn due to decay.

### 2.3. Rn Exposure Is Described by the Air Dispersion Models for the K-65 Silos

The mean annual Rn air concentrations for each sector were calculated using a gaussian air dispersion model for the K-65 silos, which is described in detail in the CDC The Fernald Dosimetry Reconstruction Project [10] (p. 62), as follows:

(1) $$X_{d,t,}(x)=K{\left(\frac{x}{\rho }\right)}^{c}\cdot \sum_{q=1}^{6}\sum_{s=A}^{F}\frac{f_{q,d,s}}{u_{q}}\cdot D_{s}(x)\cdot Qt\cdot \varphi_{resid}\cdot \varphi_{met,t}$$

where $X_{d,t}$, (x) is the ground level concentration of Rn (Ci·m^−3^) at the receptor location for direction (d) and time (t) at distance x. The terms in this model are discussed in order from left to right in the following sections.

A Rn calibration factor is used to predict Rn concentrations at the receptor locations for each calendar year. The calibration factor can be interpreted as an uncertainty distribution that moves the logarithmically plotted model curve upward (K>1) or downward (K<1) without changing the parametric shape. The calibration factor is calculated as follows:

(2) $$K\times {\left(\frac{x}{\rho }\right)}^{c}$$

where ln (K) is normally distributed with a mean of −0.44 (SD = 0.69), c is normally distributed with a mean of 0.19 (SD 0.32), x is the distance of the receptor from the emission source, and ρ is the radius of the source around the silos [10]. It is reasonable to treat the two silos as a single circular emission source with the midpoint between the two silos as the center of the emission source with a radius of 50 m. The values of the calculated calibration factors were validated against the discrete values given in Task 6 of the Fernald Dosimetry Reconstruction project report [10] (p. M-62).

The double summation term estimates the prevailing meteorological conditions obtained from the weather monitoring station at the Fernald site during a 5 year period between 1987–1991, which are provided in table ES-1 to ES-13 of the CDC Task 4 Fernald Dosimetry Reconstruction project report [4]. The cumulative monthly frequency describes how the exposure of each receptor (i.e., residential location) based on wind rose frequencies was calculated for the 12 meteorological tables in CDC Task 4, as follows:

Monthly wind frequency:

(3) $$\sum_{q=1}^{6}\sum_{s=A}^{F}\frac{f_{q,d,s}}{u_{q}}$$

where the finite double summation is the sum of two indices for the frequencies (six windspeed categories ($f_{q}$) and the six stability categories ($f_{s}$) for each direction ($f_{d}$) divided by the average windspeed ($u_{q}$) of each stability category (1, 3, 5, 7, 9, and 11 ms^−1^) [10].

Meteorological data are representative of weather conditions during the period of plant operations and were recorded at the FMPC meteorological tower, which was located about 200 m south of the production site. Prior to 1986, there were no meteorological for the years of plant operation. These meteorological data are available as joint frequency tables, with wind frequencies broken down by 6 wind speed intervals (0–2, 2–4, 4–6, 6, 8, 8–10, >10 m·s^−1^), in 16 wind rose directions. and the 6 Pasquill–Gifford stability classes, A-F, that classify atmospheric dispersion conditions related to turbulence [12,13]. Class “A” is assigned to the most unstable atmospheric conditions (most turbulent) of air and class “F” is the most stable [4] (p. G-5). Windspeed frequencies for each wind rose direction are provided for each Pasquill–Gifford stability class. For each stability class, the sum of all windspeed interval frequencies and direction frequencies total 1.0. The frequencies for each class (A–F) also sum to 1.0 for each month. The stability-adjusted wind frequency for each prevailing wind direction (i.e., NNE, SW) ($f_{q,s,d}$) is calculated as the product of each windspeed interval frequency and the frequency for each stability class. The wind ratio ($f_{q,s,d}∕u_{q}$) was then calculated as the quotient of the stability-adjusted wind frequency divided by the median windspeed (m·s^−1^), which results in a ratio with the units of s·m^−1^. Each wind ratio was summed across stability classes for each wind direction to obtain the straight-line wind rose velocity radiating from the Fernald FMPC for each month. Annualized frequencies were averaged across all months to yield the annualized frequency.

After calculating wind direction frequencies, the 16 wind rose directions were consolidated to 8 directions by splitting every other direction and combining half the wind ratios with the wind ratios of the adjacent directions. For example, the original wind rose has three north—northeasterly directions: north (N), north northeast (NNE), and northeast (NE). The direction NNE was split, and half the wind ratios were combined with N, and the remaining half with NE. In this way, the 16 directions (N, NNE, NE, ENE, E, ESE, SE, SSE, S, SSW, SW, WSW, W, WNW, NW, and NNW) were reduced to 8 directions (N, NE, east (E), southeast (SE), south (S), southwest (SW), west (W), and northwest (NW)). The total wind frequencies for the 8-direction wind rose (octet) are equal to the frequency sums for the 16-direction wind rose. The weighted wind effects were calculated as the wind frequency multiplied by the median windspeed for each windspeed class.

It is important to note that the wind directions are given as the direction from which the wind is blowing, such that a wind blowing from west to east is a westerly wind. Hence, the wind direction that blows across the source (K-65 silos) to expose northeast (NE) sectors to Rn is a southwesterly (SW) wind. Thus, the sector in which a receptor is located and the sector representing the wind direction are reciprocal (differ by 180°) [10] (p. M-10).

**Figure 2.**
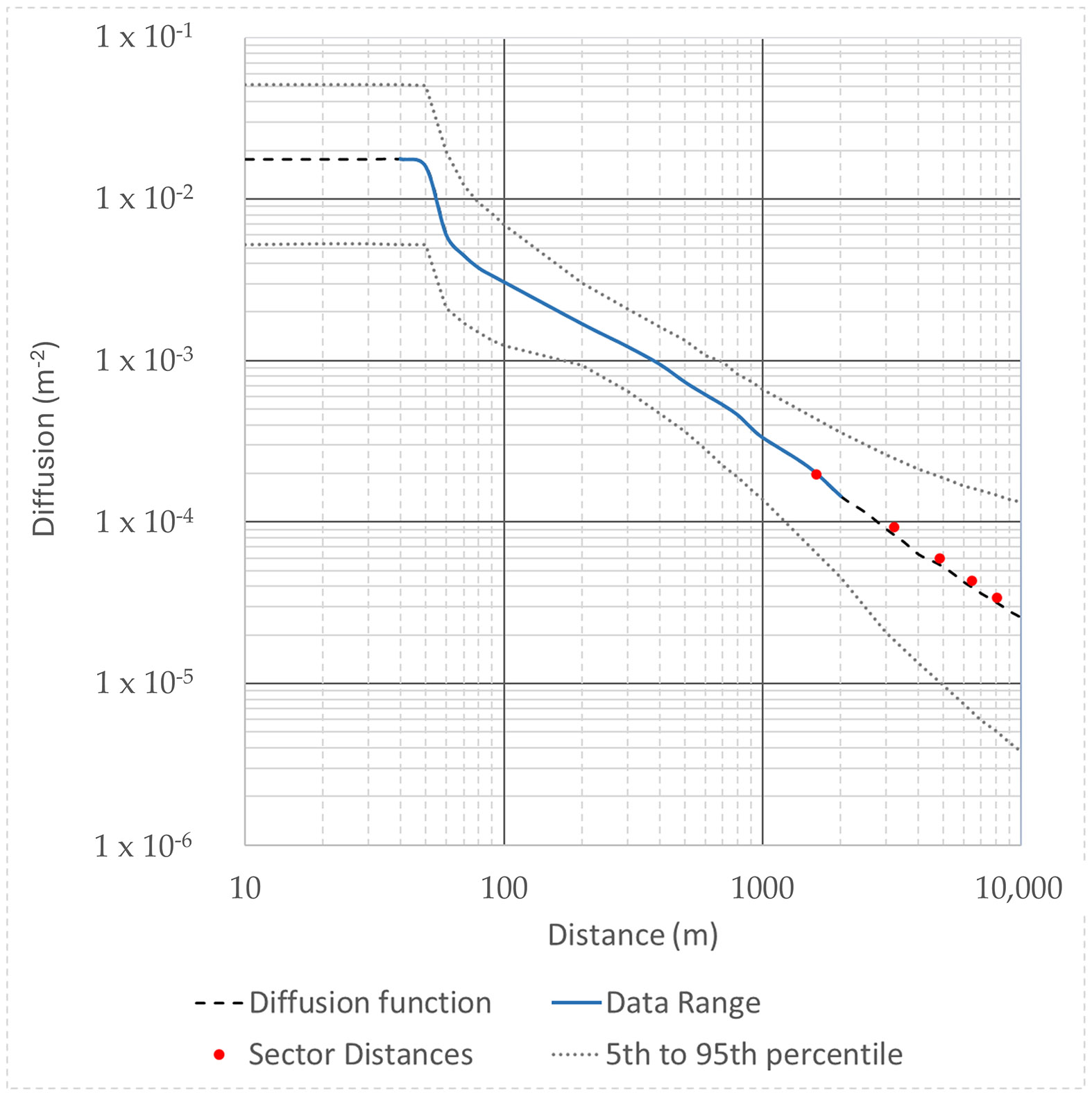
Diffusion as a function of distance for Rn from the K-65 silos assuming a Gaussian diffusion model for a ground-level circular source of 50 m. This curve is an average of the daytime and nighttime calibration curves that are based on the 1980s Rn monitoring data. Adapted from [2] (p. 43).

The diffusion function (Ds(x)) has units of m^−2^ and describes the vertical and horizontal dispersion of Rn as a function of distance from the FMPC as it moves downwind. This diffusion is described by a ground-level area-source Gaussian plume model with a circular area source with a radius of 50 m. The mathematical computation of the Rn diffusion function is complex because it is based on two relatively spare sets of air monitoring data collected in the 1980s, as well as average annualized radon emission rates for both unconstrained (pre-1980) and constrained (post-1980) daytime and nighttime silo radon release and Pasquill–Gifford atmospheric stability frequencies. To simplify our modeling process, we have adopted the diffusion model developed by the CDC Fernald Dosimetry Reconstruction project (Figure 2) [10] (p. M-24 to M-37).

Rn Emission (Qt) is the source term that summarizes the yearly release rate of Rn (Ci·yr^−1^). For estimating the Rn exposure of residents in the Fernald community, median annual Rn emissions (bounded by the 5th and 95th percentiles) were taken from the emission rates calculated by the Fernald Dosimetry Reconstruction project report [3] (p. J-69). The computation of Rn emissions from the silos during the years of FMPC operation is complicated and a very brief overview to summarize the approach is provided as Supplementary Materials. From calculated silo emissions, median release estimates were used for modeling. The estimated Rn release rate from the two K-65 silos changed seven times during the plant operations (Table 1). Every subject in a given sector received the same estimated Rn exposure for a specific year, unless the exposure time for a specific year is adjusted for change in residential status. We did not have data to adjust for partial-day changes in exposure, such as employment outside the exposure area, or when attending school outside the subject’s residential sector.

The $\varphi_{\text{resid}}$ factor is the distribution of residuals to compensate for mispredictions related to local variations such as terrain, obstructions, and nonuniform air–soil thermal interactions and has a value geometric mean (GM) = 1 with geometric standard deviation (GSD) = 1.47. The term $\varphi_{\text{met},t}$ represents the meteorological uncertainty arising from using the five-year composite meteorological dataset (based on 1987–1991) for predictions of specific years’ data with a GM = 1 and a GSD = 1.7. These last two factors are lognormally distributed multiplicative random variables where, for any non-stochastic quantity C, the product C φ is lognormally distributed with GM C and GSD equal to the GSD of φ. Since the distribution model is a discrete prediction rather than a probabilistic model, these random variables are taken to be equal to their GMs, which is 1.0 [2] (p. M-7).

The final product for the value of $X_{d,t}$, (x)is in units of Ci m^3^. This value is converted to Ci/L^−1^ (using a factor of 1000 L/m^3^). To convert this result to units comparable to the EPA advisory limit (pCi/L), a conversion factor of 1.0 × 10^12^ pCi/Ci [10] (p. I-2) is applied to yield a final Rn concentration in units of pCi L^−1^, which is directly comparable to the EPA action limit. The final value represents the annualized Rn concentration for each receptor location, based on the sector’s direction and distance from the plant.

### 2.4. Study Population, Mean, and Median Rn Exposure at Each Sector

Individual addresses were geocoded and assigned to 100 sectors radially arranged around the processing facility. The mean Rn exposure for each participant contributed by the release of Rn from the K-65 silos across all their residential addresses was calculated as the arithmetic mean of exposures for each year, and the subject resided at that address during the period from plant operation (inclusive of the period when K-65 was stored on site before plant operations officially commenced). For example, if a participant resided in sector H13 for 5 years and B03 for 7 years, the exposures for each year at sectors H13 and B03 were summed and divided by the total years of residence, which is 12 in this case. These annual estimates were adjusted for any partial years of residence.

To calculate mean individual exposures and the overall mean exposures, the arithmetic mean of mean exposures was calculated as $\left({\bar{x}}_{1}+{\bar{x}}_{2}+\ldots +{\bar{x}}_{n}\right)∕n$, where ${\bar{x}}_{1}$ to ${\bar{x}}_{n}$ represent the mean exposures of each individual participant and n is the total number of participants. To obtain complete Rn exposure histories for each participant, R software (R Foundation for Statistical Computing, v. 4.4.2) was used to first aggregate all multiple instances of the same University of Cincinnati subject identification numbers (UC IDs) for each participant’s residential location, and the period of residence at each location was used to calculate a weighted arithmetic mean of a participant’s exposure.

The mean exposure to Rn for each year (1952–1988) was calculated based on individual exposures, and then we determined which of these years exceeded the EPA action limit of 4 pCi L^−1^. To calculate the mean Rn exposure for each year, the sum of each year’s individual exposures was obtained and then used to produce an average. These averages were then compared to the action limit to determine which years had a mean exposure over the action level. The average exposure per sector over the exposure window was calculated with the aim of determining which sectors had higher average exposures to support an Rn footprint near the site. To obtain this average, R software was used to aggregate exposures for all individuals in each sector, and the mean cumulative exposures for the population of each sector were calculated. These data were used to develop and verify an Rn footprint surrounding the Fernald site. A pattern of Rn dispersion was desired to assess which directions from the site had the highest Rn exposures. Each sector falls within one of eight cardinal or intermediate directions (N, NE, E, SE, S, SW, W, and NW) of the plant. Each sector was mapped to a direction and distance relative to the plant. The sector and direction columns were aggregated from the data with over 14,000 resident addresses, which produced a list with 100 rows, one for each sector and the accompanying direction. The total number of sectors that fell in each direction was also included in the output. The Rn footprint was created from the calculation of mean exposures for each of the eight directions. This was completed by taking the sum of all exposures for each of the directions. The mean was then calculated from each of the eight sums to produce an average exposure per sector.

## 3. Results

### 3.1. Wind Velocity and Direction

**Figure 3.**
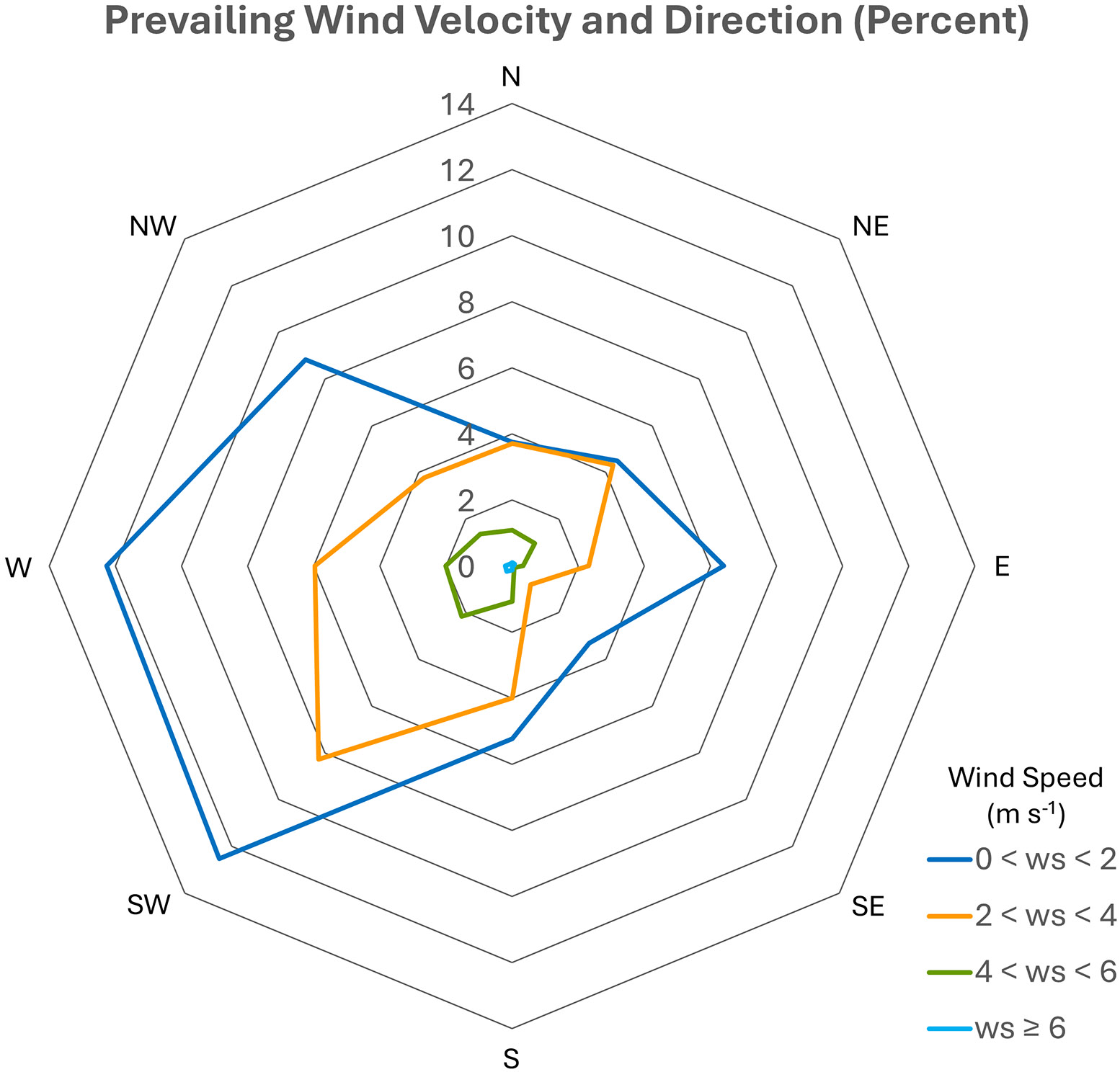
The mean prevailing wind frequencies, inclusive of all wind classifications and velocities. Frequency indicates the direction the wind is blowing. The reciprocal direction (e.g., from SE toward NW) indicated the communities exposed to Rn.

The dispersion of Rn from the silos to the surrounding communities largely depends on the average frequency of the prevailing wind direction, velocity, and stability. Previous studies involving Fernald workers have shown that the highest exposures to Rn occurred in the southeast and northeast directions [14], which suggests that westerly winds were most frequent. Wind directions were assumed to be the same, on average, over the years of plant operations. The wind direction was classified by the direction from which the wind was blowing. For example, if the prevailing wind was from the south westerly direction, it was across the silos and toward residential sectors in the direction that is the reciprocal of the prevailing wind northeast of the plant. In general, prevailing winds in the region of southwest Ohio are most frequently from the northwest, west, and southwest directions, based on the octet wind rose used for this study. On an annualized basis, the most frequent prevailing wind speeds were within the <2 m·s^−1^ wind classification (Figure 3), and the frequency of wind velocities greater than 6 m·s^−1^ was very infrequent. Although Pasquill classifications are included in the meteorological data used for exposure modeling, these classes are not applied in this analysis; instead, the double sum procedure (Equation (3)) reduces this dimension such that only the annualized prevailing wind frequencies, velocities, and directions influence exposure estimates.

**Figure 4.**
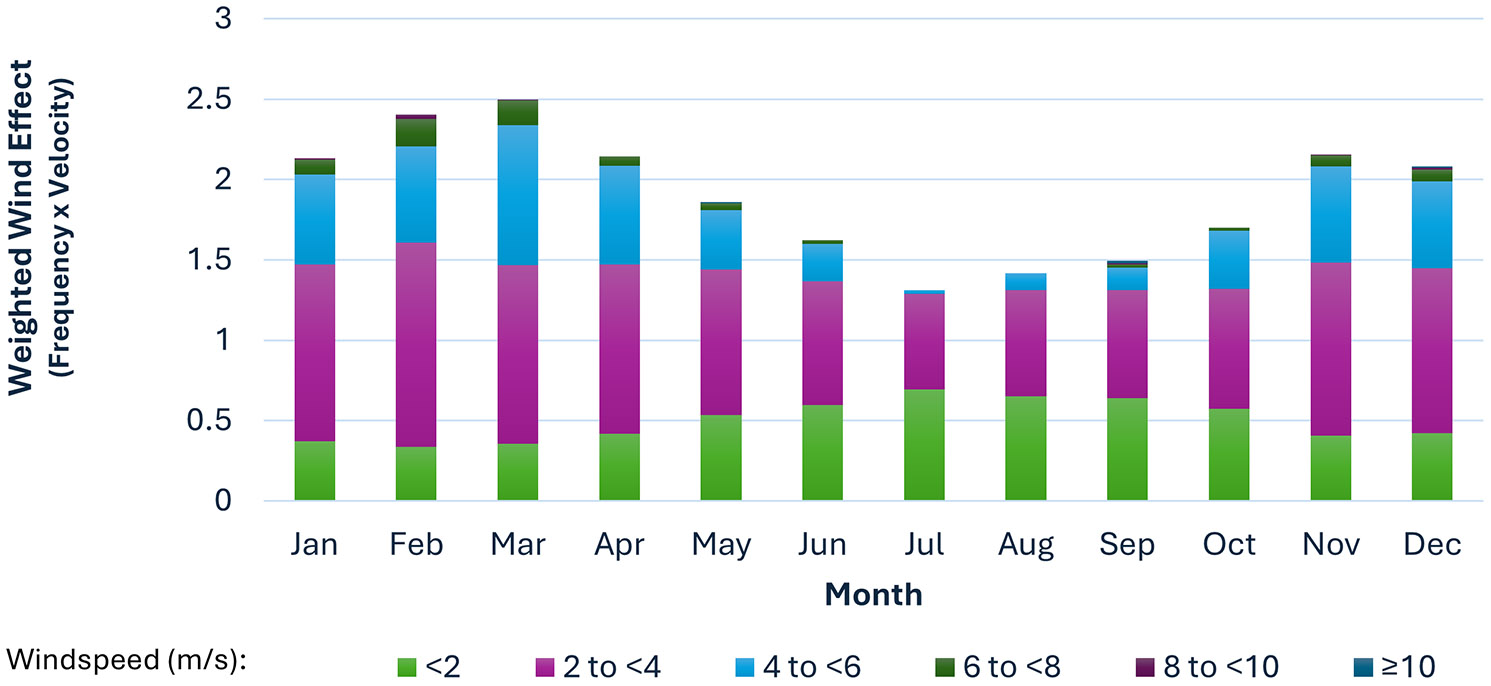
Annual frequency-weighed wind velocities (wind power) across all directions and stability classes.

Wind velocities were assumed to be constant on an annual basis for the estimate of Rn exposure. This simplifying assumption was applied to any subject where the residential location was for a full year. However, most subjects in the study area had partial year occupancy at the start and end of their residential period. This is to be expected since it is uncommon for people to move into a residence at the beginning of January and move out at the end of December. For people with partial year exposures attributable to residential relocations, monthly fluctuations in wind speed and frequency for each octet were used for exposure estimation. The monthly fluctuation in frequency-weighted wind velocities is summarized in Figure 4.

### 3.2. Population Distribution Changes

**Figure 5.**
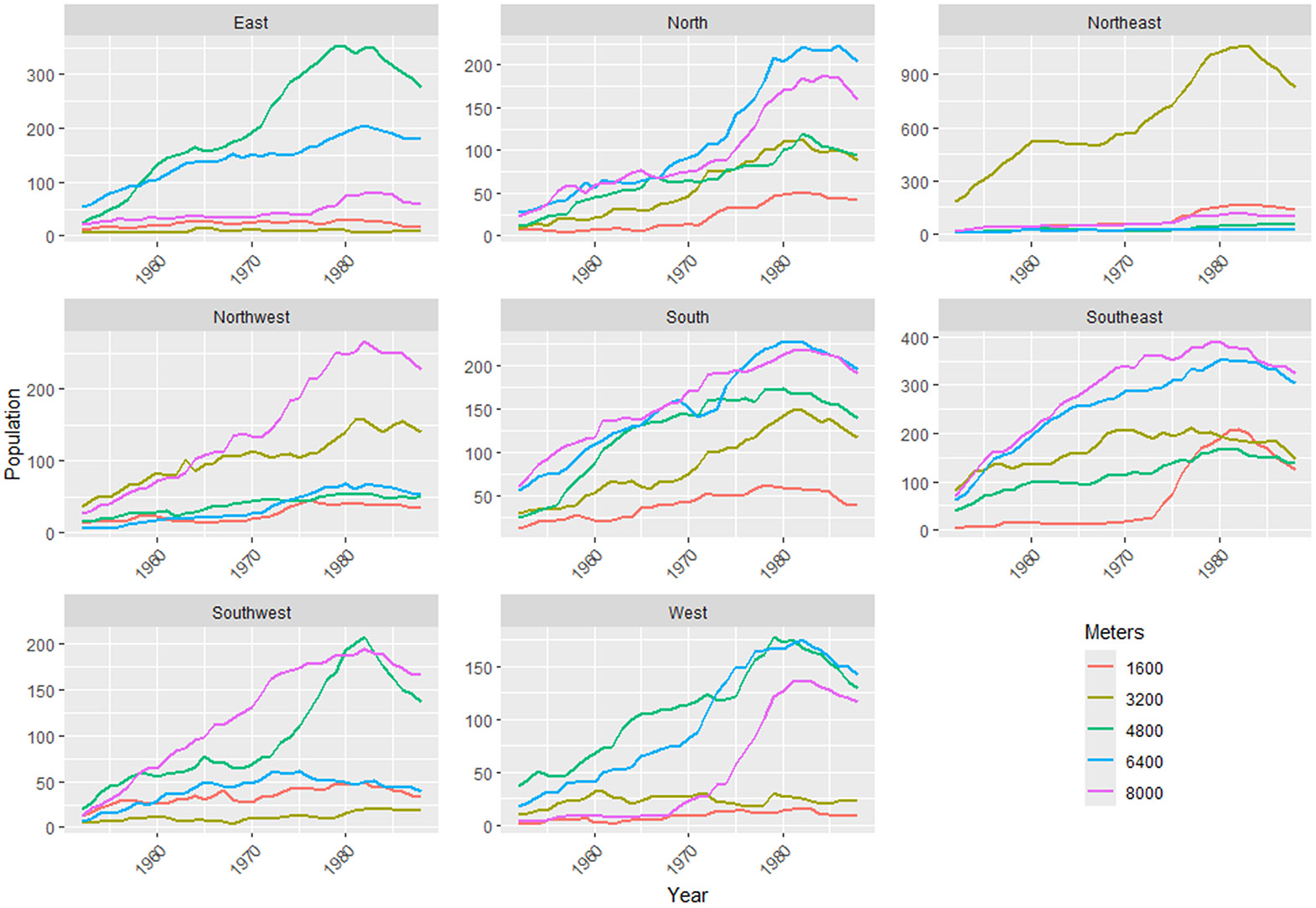
Mean overall populations residing within an 8 km radius of the FMPC for each octet direction and distance over the period of FMPC operation.

The spatial distribution of where cohort residents lived during the period of plant operation is important for characterizing those with the greatest health risk from Rn exposure. To start with, we looked at the total number of residents within the study area (8 km radius) from the start of plant operations in 1951 through to the end of operations in 1988. The population increased up to the 1980s, after which a decrease in population was noted (Figure 5). The decreased population corresponds with the decommissioning of the plant in the 1980s.

Over the decades of plant operation, the population surrounding the FMPC changed in size and distribution. The changing pattern of the population residing in the area surrounding the FMPC shows that sectors to the northeast, east, and southwest are the most densely populated and demonstrate the greatest growth in population over time (Table 3).

### 3.3. Mean Rn Exposure per Year

**Figure 6.**
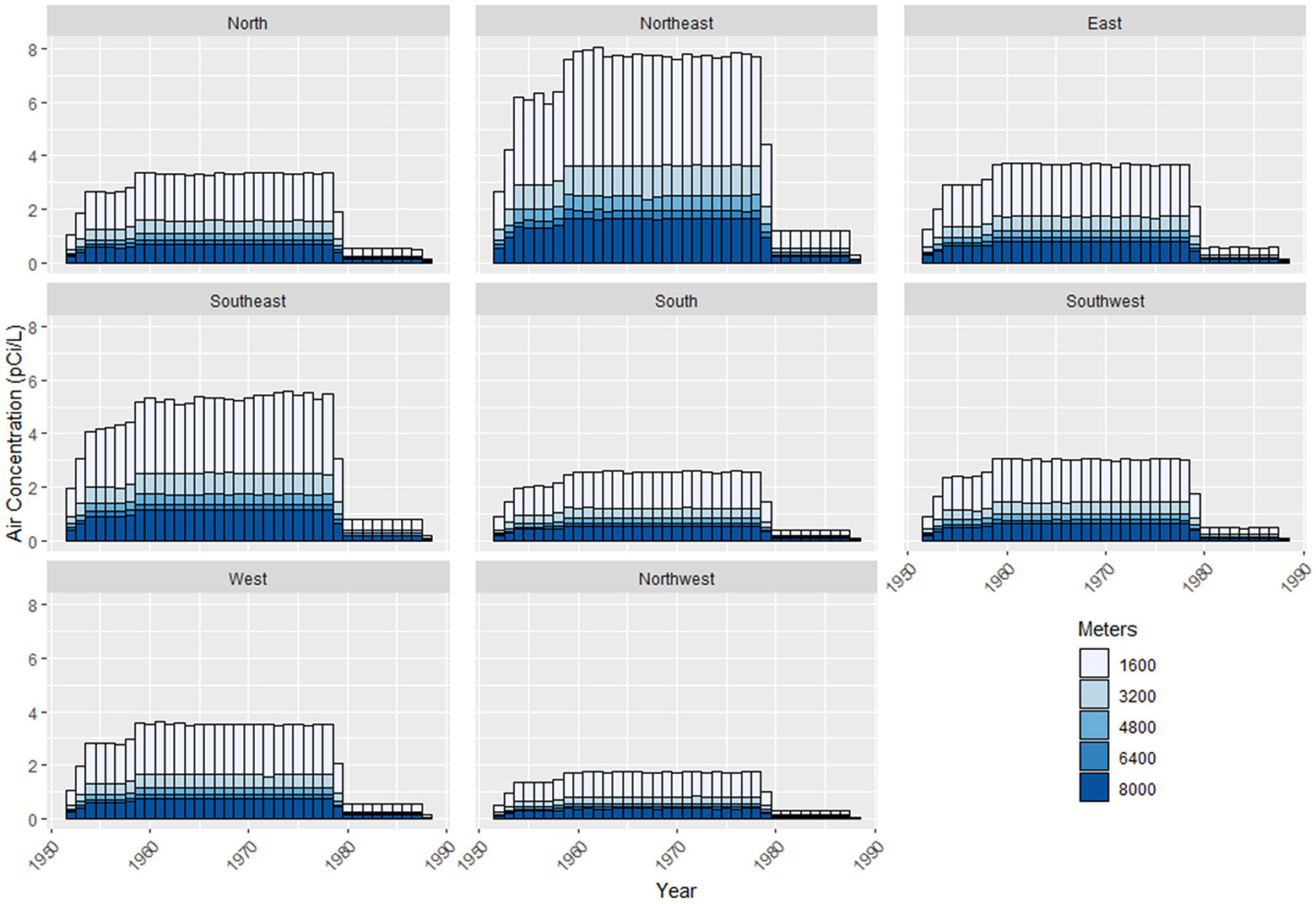
Bar plot for mean Rn exposures by octet direction and distance over the period of FMPC operation.

The magnitude of annual Rn exposures by residential location is summarized by year (1951–1988), distance, and direction from the plant (Figure 6). This comparison represents the exposure for each participant at each residence without accounting for multiple residential locations, which can be in different sectors during the period of exposure.

The 10 sectors with the highest average Rn exposures across all years of FMPC operations are listed in Table 4. Sector B09 was the sector with the highest population and was third overall in terms of Rn exposure, which means that this sector had the greatest exposure-related risk per capita across all sectors.

Rn exposures were estimated for participants at each residential address. Every address was assigned to a sector that received an estimated amount of Rn during a specific year. Everyone who resides in a sector over a full year is assumed to have received the same annual dose for that specific year. As noted above, the amount of Rn fluctuated depending on the rate of Rn release from the FMPC silos. The aggregated mean annual Rn exposures (pCi L^−1^) for all individuals, calculated from each individual’s mean exposure at all residences within the study area, are summarized in Table 5.

### 3.4. Number of People with Exposure That Exceeded the EPA Action Level

The number of individuals with mean Rn exposures exceeding the EPA action limit was calculated based on the average exposure for each subject during their full window of exposure. This approach accounts for cases where some individuals may have resided in multiple sectors at any time during the 37-year period of FMPC operation. The fraction of individuals with annualized Rn exposures exceeding the action limit of 4 pCi L^−1^ for any single year was 1.67%; however, this is likely to be an undercount because it relies on exposures averaged over the full year, and it is likely that weather conditions exposed some individuals to Rn concentration greater than the 4 pCi L^−1^ action limit during some months of the year but, on average over the full year, exposure were below the action limit. Based on prevailing wind patterns, elevated exposures to communities located northeast, east, and southeast of the plant occurred during the period from June to October. During these months, wind velocities are generally lower, resulting in less dilution of the Rn plume and higher air concentrations. During the peak years of Rn release from 1954 to 1979, residents in sectors within 1600 m of the plant boundary and to the northeast, east, and southeast had the highest predicted exposures. During these years, 240 subjects residing in sectors B03, 31 in B08, 612 in B09, 25 in H01, and 229 in H02 had mean annual Rn exposures exceeding 4 pCi L^−1^.

## 4. Discussion

**Figure 7.**
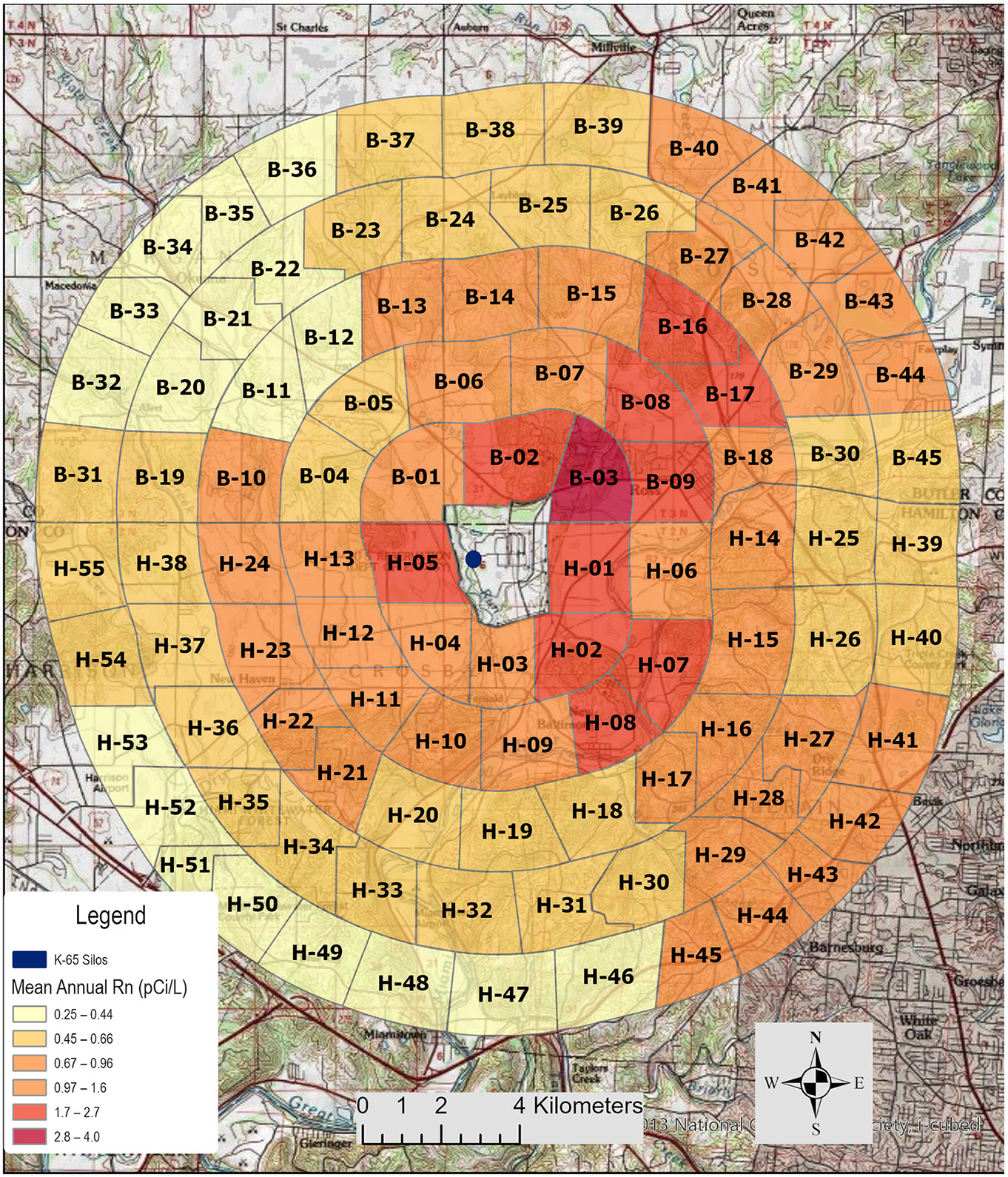
Visualization of the sectors with the mean Rn exposure estimates over all years of FMPC operations. In this figure, north is the top of the Figure, and the most highly exposed sectors are to the northeast, east, and southeast directions. The sectors directly to the west of the plant are closest to the K65 silos.

Rn was released from the K-65 storage silos located at the Fernald FMPC from the year 1952 through to 1988. This emitted Rn is in addition to Rn levels naturally occurring in this region of Ohio, which have been reported to range between 0.24 pCi L^−1^ [15] and 0.58 ± 0.17 pCi L ^−1^ [16], depending on the location and study. Over these years, the population surrounding the plant grew, and some individuals changed residential locations during the period of plant operations, which affected their Rn exposure. We have evaluated annual Rn exposures for each member of the Fernald Community Cohort across all places of residence within the exposure area during the years of plant operations. In 1998, the Centers for Disease Control and Prevention (CDC) estimated lung cancer mortality risk for individuals living within 10 km of the Fernald FMPC [14]. Table 2 of the CDC Lung Cancer report listed median lifetime lung dose equivalents for Rn released from the FMPC at distances of 0–4, 4–7, and 7–10 km in the NE, SE, SW, and NW directions. The population with the highest lifetime equivalent dose of Rn resided in the southeast of the FMPC at 0–4 km (approximately 0–2.5 miles) and had a median value of 1.44 Sv. The second highest Rn lifetime lung dose was noted to be 0.86 Sv in the NE direction, also at approximately 0–2.5 miles from the center of the site [14]. The median Rn lung doses reported for the NW and SW directions were much lower in comparison. We have shown that the highest Rn exposures occur in the NE, E, and SE directions. This finding is similar to the CDC lung cancer report but differs slightly in that the CDC report, which used a wind rose classification system consisting of 16 directions centered on the plant and was aggregated into only 4 cardinal directions, whereas the FCC study used 8 directions [11]. The different wind rose systems result in some differences in how we classified sectors compared to CDC domains, but the overall results are similar. The dispersion of Rn is concordant with prevailing wind patterns in the Fernald area (Figure 3) such that the sectors with the highest exposures are downwind of the facility and closest to the fence line of the facility (Figure 7).

Previous dose reconstruction efforts have characterized Rn exposures to employees who worked at the Fernald FMPC during its years of operations. A total of 7143 Fernald workers between 1952 and 1988 had working level month (WLM) doses determined based on combined estimates from K-65 and Q-11 ore silos sources of exposure [17]. The arithmetic yearly mean (AM) for workers from the K-65 silos ranged from 0.17 to 0.99 WLM (0.014 to 0.082 WL) between 1952 and 1979, separate from the estimated exposure from the small Q-11 source [17]. The emissions from the Q-11 ore silos were much less than those of the K-65 silos and primarily impacted workers; the relative contribution of these the Q-11 silos to the overall Rn exposure of the surrounding community was very small compared to the emissions from the K-65 silos. For comparison, the 90% probability interval (5th to 95th percentile) emission form the Q-11 silos ranged from 30 to 200 Ci y^−1,^ compared to 3100 to 7600 Ci y^−1^ for the K-65 silos [3] (p. J-20).

The objective of this project was to evaluate the Rn exposure of the Fernald community surrounding the FMPC. This information, together with participant health-related information, will enable future investigations of the role of Rn on lung cancer incidence in the Fernald Community Cohort. The current work relies on a discrete model of Rn exposure as a starting point for evaluating the relationship of Rn with lung cancer occurrence in the Fernald area. However, there is a large amount of uncertainty that is not captured in this discrete model. Depending on the findings from the pending health effects analysis, there may be a strong justification for transforming the deterministic model used here to a probabilistic Monte Carlo analysis to characterize the interrelationships of exposure uncertainties in all aspects of the model and these exposures to outcomes.

The study domain around the FMPC is in a semi-rural area with many small and large farms where individuals spend significant portions of their time outside. Rn from the K-65 silos also would have entered homes. Under usual circumstances, when Rn enters homes from geologic sources, ventilation allows Rn to escape and limits the deposition of Rn daughter nuclides. However, when the source of Rn is outdoor ambient air, a well-ventilated home provides minimal protection from Rn exposure. In the timeframe of the FMPC operation, a typical home had ventilation rates air changes/hour) in the range of 0.2 to 3 h^−1^ [3,18]. In the presence of persistent Rn levels, the Rn levels of the air inside many homes (and other buildings) would likely rapidly equilibrate with Rn levels in ambient air released by the FMPC. In fact, the ventilation rates of a typical home (50th percentile is 0.40 h^−1^) is much greater than the ventilation rates of the K-65 silos, which range from about 0.002 h^−1^ to 0.05 h^−1^, depending on the year [3,10]. For these reasons, the difference in Rn content between indoor air and outdoor air is minimal.

The average concentration of Rn in homes in the United States is about 1 pCi L^−1^ [19]. The risk associated with exposure to this concentration is about 0.5% of a lifetime [10]. EPA has established an action limit for Rn of 4 pCi L^−1^ [9]. An action limit is the concentration above which action should be taken to reduce the Rn concentration as low as is reasonably possible, which is typically a value set by the EPA [20]. However, in contrast to occupational exposure limits, the action limit should not be considered a “safe” exposure. For Rn, as with other mutagenic agents, the EPA considers there to be no known safe level of exposure. In fact, the EPA has recommended that Rn levels of 2 pCi L^−1^ or greater should be reduced, and the United Nations World Health Organization (WHO) recommends a reference level of 2.7 pCi L^−1^ (100 Bq/m^3^) [21,22]. Some individuals in the highest exposed sectors exceeded the EPA 2 pCi L^−1^ recommendation and are in the range of the WHO recommended limit. Many sectors were found to exceed the amount of Rn typically found in outdoor air (0.4 pCi L^−1^).

Although a few of the individual exposures exceeded the EPA action limit based on the result of the current annual exposure model, the exposures for individuals in some sectors are substantial, especially in the early decades of plant operation, and these predicted exposures are likely well below the worst-case scenario for several reasons. As noted above, the discrete model used here uses median estimates for most of the model parameters and annualized weather parameters. Using 75th or 95th percentile estimates substantially increases exposure estimates. Since Rn release and dispersion are largely a function of weather conditions, it is likely that individuals in some sectors receive much higher Rn exposure on some days during the year, particularly during the months of May through October when silo heating and prevailing winds carry Rn toward neighboring sectors. These factors should be investigated in future Monte Carlo models. Reanalysis of the available data based on the upper confidence limits for Rn release or using monthly meteorological data would show that the action level could reasonably have been exceeded at some points during the years of FMPC operation. Since Rn release is on a log scale, the release rate that is double the median rate is still within the 95th percentile confidence band. Exposure to Rn has historically been linked to an elevated risk of lung cancer development [23]; however, lung cancer incidence in the FCC has not been evaluated to date. There is one study that has projected a possible 1% to 12% increase in lung cancer mortality across all residents residing within 10 km (6.2 miles) of the FMPC based on estimates of combined exposures to radon, uranium, thorium, and their decay products [14]. Here, our determination of atmospheric Rn exposures among Fernald residents links exposure estimates to individual residents in the FCC medical monitoring data, which will enable a much more robust analysis of lung cancer risk.

Rn concentration in air is inversely related to wind velocity; the higher the wind velocity is, the more diluted the Rn concentration becomes (assuming the release rate is not influenced by wind speed), and the more an emitted gas or dust will spread over time, which reduces the air concentration. As shown in Figure 4, from January to March, as well as November and December, wind frequencies and velocities are relatively higher; conversely, during April through October, wind velocities are lower, with a nadir in July. During warmer months, when gentler wind speeds are more frequent, Rn concentrations are elevated. The opposite is true during the winter months, when the frequency of higher velocity winds increases, resulting in a greater dilution of Rn and further spread from the emission source. This results in lower concentrations for each sector. Additionally, wind during nighttime tends to be calmer than during the day, and the rate of Rn release from the silos is lower due to reduced thermal pumping and calmer winds [10].

There are several limitations to the current analysis of Rn exposure. One of these limitations is that we do not have workplace or school information on participants, which could influence individual Rn exposure estimates. None of the individuals in the cohort worked at the FMPC, as the workers had a separate medical monitoring program; however, some residents may have worked at locations outside the study area, which would mitigate the estimated exposures. Likewise, children attended school for about 6 h per day, 9 months per year, which could influence exposure, depending on the location of the school. These factors are important because Rn release from the K-65 silos was greater during the day than at night for most of the time that the plant was in operation [10]. Incorporating work and school sites for cohort participants could add greater certainty to the exposure assessment. A second limitation of the current analysis is that it does not include exposure estimates for daughter nuclides produced by the decomposition of Rn. In part, this choice was driven by a desire to focus on Rn emissions for comparison to guidance exposures levels and by the absence of information about the fractional equilibrium of short-lived ^222^Rn daughters in silo air [3]. Thirdly, because Fernald is located in a very rural community, there is no meteorological data from the plant site for most years of operation. The meteorological data used here were collected during the period when the plant was decommissioned and during remediation of the FMPC site. These data are considered to be representative of weather conditions during plant operations.

## 5. Conclusions

Prolonged Rn exposures have been previously associated with increased lung cancer incidences [7,23]. Future studies of Rn exposures to the members of the Fernald Community Cohort could make comparisons between the incidence of lung cancer or other diseases associated with Rn estimates to the Fernald community. We concluded that of the 9318 subjects that resided in the study area circling the Fernald FMPC between 1952 and 1988, 1019 received at least one mean yearly Rn exposure of greater than 4 pCi L^−1^. It is likely that if monthly exposures were considered, many more subjects would have had monthly exposures exceeding the EPA action limit. In this respect, a total of 3059 subjects are predicted to have had Rn exposures exceeding the EPA recommendation level of 2 pCi L^−1^. The Rn footprint (Figure 7) shows that the highest mean levels are in sectors in the northeast, east, and southeast directions.

## Supplementary Material

Supp. data

**Supplementary Materials:** The following supporting information can be downloaded at https://www.mdpi.com/article/10.3390/atmos16080939/s1: Supplementary S1: A brief description of the procedure used to estimate 222Rn emission from the FMPC K-65 silos.

## Figures and Tables

**Table 1.** Events influencing the release of Rn from the Fernald FMPC K-65 silos.

Event	Years		Rn Release (Ci·y^−1^) ^‡^		
	Start	End	Median	5th%	95th%
Silo1 actively filled	Jul ‘52	Jun ‘53	1900	200	4200
Silo2 actively filled	Jun ‘53	Sep ‘58	4900	3100	7600
Before sealing silo domes	Sept ‘58	Jun ‘79	6200	4200	8700
After sealing silo domes	Jul ‘79	Dec ‘87	950	360	1700
Foam insulation of silo airspace	Jan ‘88	Dec ‘88	540	120	1300

Adapted from [2] (p. 26). ^‡^ Joint Rn release from both operational silos [3] (p. J-69).

**Table 2.** Summary of the total number of residential histories with missing information.

		Population (n)	Number of Residential Histories Missing:			
			From Month	To Month	From Year	To Year
Residences	Number	14,658	1244 *	1107 **	72 ^‡^	106 ^‡^
	%	100	8.49	7.55	0.49	0.72
Participants ^†^	Number	9375	892	766	57	91
	%	100	9.51	8.17	0.61	0.97

* The date 1 July is assigned as the default “From” month when not reported by participant.

** The date 30 June is assigned as the default “To” month when not reported by the participant.

‡ Residences without “From” or “To” years were removed from the analysis.

† Participants who are affected by missing residence information.

**Table 3.** Ten most populous sectors during the 1950s and 1980s.

Sector	Octet	Population 1950s	Sector	Octet	Population 1980s
B09	NE	566	B09	NE	1297
H08	SE	128	B18	E	327
B05	NW	92	H02	SE	289
H26	E	90	H27	SE	211
H28	SE	82	B05	SW	198
H27	SE	77	B03	NE	195
H17	SE	71	B37	N	189
H41	SE	69	H22	SW	186
B18	E	66	H08	SE	185
H07	SE	63	B36	NW	163

**Table 4.** Mean annual Rn exposures (pCi L^−1^) for the 10 highest sectors.

Sector	Octant	Distance (m)	Mean (pCi L^−1^)	Maximum (pCi L^−1^)
B03	NE	1600	3.96	5.49
H02	SE	1600	2.73	3.78
B08	NE	3200	2.36	3.27
B09	NE	3200	2.36	3.27
H01	E	1600	1.89	2.61
H05	W	1600	1.81	2.51
B16	NE	4800	1.78	2.46
B17	NE	4800	1.78	2.46
B02	N	1600	1.71	2.37
H07	SE	3200	1.63	2.26

**Table 5.** Summary of residential air Rn concentrations (pCi L^−1^).

Population (n)	Arithmetic Mean ± Std	Geometric Mean ± Std	Median	95th Percentile	99th Percentile	Maximum Annual
9318	1.14 ± 1.09	0.76 ± 2.54	0.75	3.42	5.16	8.90

## Data Availability

The data used in this study are available from the Fernald Community Cohort in accordance with the Data Access protocol. That protocol and the application for data are available at https://med.uc.edu/depart/eh/research/projects/fcc/sharing-of-fcc-data-and-biospecimens. Since the combination of values of the variables will identify a person in this geographical population, no data, including nonidentifiable data, can be made available without the consent of the UC IRB.

## Abbreviations

The following abbreviations are used in this manuscript:

Bq: Becquerel
CDC: Centers for Disease Control and Prevention
Ci: curies
DOB: date of birth
E: east
EPA: U.S. Environmental Protection Agency
FCC: Fernald Community Cohort
FMPC: Fernald Feed Materials Production Center
GM: geometric mean
GSD: geometric standard deviation
L: liter
m^3^: cubic meters
N: north
NE: northeast
NW: northwest
pCi: picocuries
Rn: radon
S: south
SAS: Statistical Analysis System
SE: southeast
SV: Sievert
SW: southwest
UC ID: University of Cincinnati subject identification number
W: west
WHO: United Nations World Health Organization
Y: years
