# Supplementary material for: Radon Exposure to the General Population of the Fernald Community Cohort
Source: Atmosphere (Basel). Author manuscript; Available in PMC 2025 Sep 19. (PMC12445060; doi:10.3390/atmos16080939)
Supplement: Supp. data [file NIHMS2108960-supplement-Supp__data.pdf]

atmosphere

Supplemental Materials

Radon Exposures to the General Population of the

A brief description of the procedure used to estimate 222Rn emission from the FMPC K-65 silos

The estimation of ^{222}Rn release is discussed in detail in the Fernald Dosimetry Reconstruction Project report by Voilleque et al, 1995, Appendix J [1]. The full procedure is complicated, and a detailed discussion is beyond the scope of this paper; however, a high-level overview is provided as background. The estimate of radon release from the FMPC silos must account for several factors over the period of plant operations, including evolving structural changes that occurred with the silos, changes in silo contents during the 1950s, and very limited data on ^{222}Rn concentrations in the silos, release rates or atmospheric air monitoring data. ^{222}Rn releases from the K-65 silos were developed at a time resolution of one year. There are no direct measurements of the release of ^{222}Rn (or daughter products) from the silos. In addition, until the 1980s there were no measurements of parameters that can be used to indirectly calculate radon releases. However, because the ^{222}Rn concentrations in the sealed silos were measured during the 1980s, these ^{222}Rn concentrations were used for estimating releases rather than using the characteristics of the silo contents, which introduces numerous uncertainties.

The method that was used to estimate Rn release considers two key time periods, before 1980 and after 1980, and two emission mechanisms: air exchange (Q_{exch}) and diffusion through the silo structure (Q_{def}). Release estimates take into consideration variables that include silo contents, structure, temperature cycling, filing history, structural modification history, air exchange rates and silo permeation rates. The release rate after 1980, when the domes were sealed, can be calculated from the product of Rn concentrations in the silo air space, the silo ventilation rate or diffusion rate constant, and the airspace volume in the silo. The effects of temperature and pressure were also important during this period because it was determined that the sealed silos could not hold pressure, and changes in temperature resulted in volumetric changes in silo air, which released excess volume to the atmosphere (and vice versa with cooling). Hence, the silo ventilation rate is the sum of a ventilation rate after 1980 is attributed to the temperature effect (“thermal pumping”). Diffusion is attributed to cracks and pores in the silos dome, and to a lesser extent (especially after the addition of the berms) the silo walls.

There are no measurements of the Rn air concentrations or silo ventilation rates before the silos were sealed in 1980. During the period of 1959 to 1979, emission estimates require an

estimate of the Rn production rate (P_{Rn}), which is the release of Rn from K-65 material into the silo air. P_{Rn} prior to 1980 was calculated from the Rn release rates and air concentrations measured after 1980. The production rate estimate is indirectly supported by the presence of short-lived daughters of ^{222}Rn, which are at concentrations close to equilibrium in the material in the silos. These daughter products emit gamma radiation, which are measured as exposure rates on the silo domes and correlate with Rn production. Rn production rate, together with air exchange and diffusion rates provide estimates of Rn release between 1959 and 1979. Prior to 1959, weighting factors are applied to account for differences in operating status, which include the addition of drummed materials, high-grade pitchblend and ore process at the plant. In 1988, additional sealing materials were applied to the silos and a modification of the method used for the period of 1980 to 1987 was used to estimate release in 1988.

It is noted that Appendix J of the report by Voilleque et al. (1995) report also includes alternative modeling procedures that were used to benchmark the release estimates from the preferred model, including an unconstrained model (assumes that Rn release from the K-65 material is not constrained by concentrations of Rn in the silo air) and a traditional method, similar to the approach used for estimating Rn release from soil-like matrix into the air.

1. Voilleque, P.G.; Meyer, K.R.; Schmidt, D.W.; Rope, S.K.; Killough, G.G.; Case, M.; Moore, R.E.; Shleien, B.; Till, J.E. *Tasks 2 and 3: Radionuclide Source Terms and Uncertainties*; The Fernald Dosimetry Reconstruction Project:; U.S. Centers for Disease Control and Prevention, 1995;URL:https://stacks.cdc.gov/view/cdc/142455.
